# *Haemaphysalis longicornis* HSP20 inhibits *Rickettsia heilongjiangensis* replication by targeting the pathogen 50S ribosomal protein

**DOI:** 10.1186/s13071-026-07398-x

**Published:** 2026-04-08

**Authors:** Yuchao Zhang, Lian-Feng Li, Wen-Jie Zhu, Tingwei Pei, Han Wang, Xiujie Liang, Yunsheng Tang, Chunyuan Wang, Songbo Zhang, Wu-Chun Cao, Zhijun Yu, Tianhong Wang

**Affiliations:** 1 Department of Biochemistry and Molecular Biology, College of Basic Medicine, Hebei University of Chinese Medicine, Shijiazhuang, 050200 Hebei People’s Republic of China; 2 Hebei Key Laboratory of Animal Physiology, Biochemistry and Molecular Biology, Hebei Collaborative Innovation Center for Eco-Environment, College of Life Sciences, Hebei Normal University, Shijiazhuang, 050024 Hebei People’s Republic of China; 3 Beijing Institute of Microbiology and Epidemiology, Beijing, 100071 People’s Republic of China

**Keywords:** *Haemaphysalis longicornis*, *Rickettsia heilongjiangensis*, Heat-shock protein 20, RNAi, Yeast two-hybrid

## Abstract

**Background:**

The tick *Haemaphysalis longicornis* is a major vector for several zoonotic pathogens, including *Rickettsia heilongjiangensis*. Small heat-shock proteins (sHSPs) are critical for stress responses and host–pathogen interactions. Among them, the *HSP20* gene was found upregulated during rickettsial infection, whereas its specific function at the host–pathogen interface remains undefined.

**Methods:**

The full length of the *HSP20* gene (*HlHSP20*) and its expression profile was characterized in *H. longicornis*, and RNA interference (RNAi) was used to knockdown *HlHSP20*, followed by the quantification of *R. heilongjiangensis* proliferation. Proteins from *R. heilongjiangensis* interacting with HlHSP20 were identified using GST pulldown coupled with liquid chromatography–tandem mass spectrometry (LC–MS/MS) and validated by yeast two-hybrid (Y2H) assays.

**Results:**

HlHSP20 is a conserved, non-transmembrane intracellular *sHSP* with a αB-crystallin domain, showing the highest expression in egg and larval stages. The knockdown of HlHSP20 significantly promoted the proliferation of *R. heilongjiangensis*, and interaction screening revealed that HlHSP20 specifically binds to the 50S ribosomal protein L14 (RhRPL14) of *R. heilongjiangensis*.

**Conclusions:**

The present study demonstrates that HlHSP20 acts as a host restriction factor against *R. heilongjiangensis* in *H. longicornis*, likely through a direct interaction with the pathogen ribosome protein. This work unveils a novel component of the antimicrobial defense of ticks and identifies HlHSP20 as a potential target for disrupting rickettsial transmission.

**Graphical Abstract:**

**Figure:**
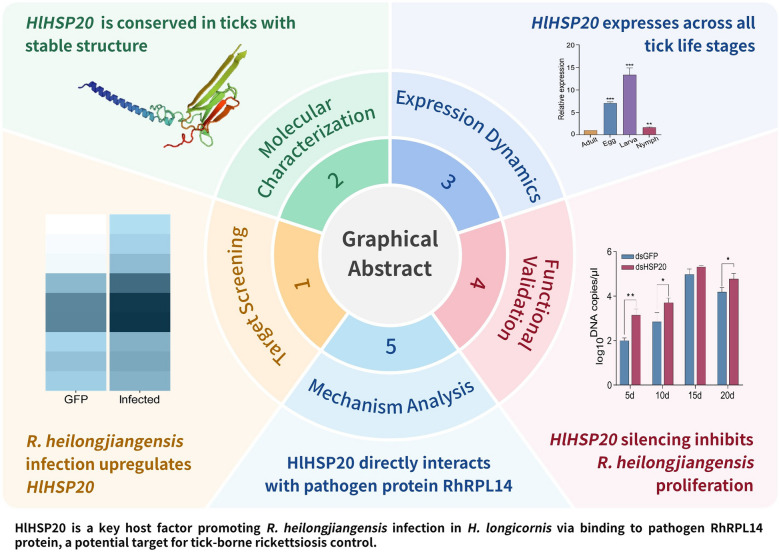

**Supplementary Information:**

The online version contains supplementary material available at 10.1186/s13071-026-07398-x.

## Background

Tick-borne rickettsioses represent a growing global health concern, primarily driven by the expanding geographic distribution of tick vectors and their capacity to transmit diverse pathogens within the spotted fever group Rickettsiae (SFGR) [1, 2]. Among the principal vectors, *Haemaphysalis longicornis* plays a critical role, particularly in the transmission of *Rickettsia heilongjiangensis*, the causative agent of far-eastern spotted fever (FESF) [3, 4]. Although the clinical manifestations and public health impact of this zoonosis are well-recognized, the molecular mechanisms governing the interaction between *H. longicornis* and *R. heilongjiangensis*, particularly the host factors influencing pathogen establishment and replication, remain poorly defined [5, 6]. Elucidating these host determinants is paramount for understanding the vectorial capacity of *H. longicornis* and for developing targeted control strategies.

*Rickettsia* species are obligate intracellular bacteria that have evolved sophisticated strategies to colonize and replicate within arthropod hosts. Their survival and proliferation are critically dependent on the cellular environment and metabolic resources of hosts [7]. Consequently, host cellular proteins that directly or indirectly influence pathogen life cycle progression are key determinants of the host–pathogen relationship. Small heat-shock proteins (sHSPs) are a conserved family of molecular chaperones known to participate in protein homeostasis, cellular stress responses, and protein folding [8, 9]. The sHSPs are evolutionarily conserved molecular chaperones that contribute to development, cellular stress tolerance, and increasingly, innate immunity and host–pathogen interactions across arthropods [10, 11]. However, their specific roles in SFGR infection within ticks remain poorly characterized.

Notably, recent transcriptomic analyses revealed the upregulation of *HSPs* in *H. longicornis* following infection with *R. heilongjiangensis* [12]. Among these upregulated genes, *H. longicornis* HSP20 (*HlHSP20*) was identified as a member of this response. However, limited information was known regarding its specific role in tick-SFGR interactions. While *sHSPs* are generally implicated in cellular defense and stress tolerance [9, 10], the precise function of *HSP20* in *H. longicornis* and its specific contribution to mediating resistance against *R. heilongjiangensis* have not been experimentally determined. Addressing this knowledge gap is critical for understanding how ticks manage intracellular pathogen infections.

To experimentally determine the function of *HlHSP20*, this study systematically investigated its role in the host–pathogen interaction with *R. heilongjiangensis*. The gene was cloned and functionally characterized, along with its encoded protein. Its expression profile was assessed across different developmental stages of the tick. The role of *HlHSP20* in the proliferation of *R. heilongjiangensis* was further investigated via RNA interference (RNAi)-mediated knockdown. To identify potential interacting proteins of *HlHSP20* from *R. heilongjiangensis*, protein–protein interaction assays were performed, including GST pulldown and yeast two-hybrid (Y2H) screening. By clarifying the molecular mechanisms through which *HlHSP20* influences host–pathogen dynamics, this research provides crucial insights into tick resistance to SFGR and lays the groundwork for potential targeted interventions.

## Methods

### Tick rearing and *Rickettsia* culture

Parthenogenetic *H. longicornis* ticks were collected from Cangxi County, Sichuan Province, China, via the flag-dragging method. Ticks were immediately transported to the laboratory. During parasitic feeding, ticks were maintained on the ears of New Zealand white rabbits. For nonparasitic stages, ticks were reared in an incubator at 26 ± 1 °C, 85% relative humidity, with a 16:8 h light/dark photoperiod. The *R. heilongjiangensis* strain (GenBank accession: GCF_035117685), isolated from field-collected *H. longicornis*, was characterized in our laboratory. Stock cultures were stored in Dulbecco’s modified Eagle medium (DMEM) with 20% fetal bovine serum (FBS) and 10% glycerol at −80 °C. All animal procedures were approved by the Institutional Animal Care and Use Committee (IACUC) of the Beijing Institute of Microbiology and Epidemiology (approval no. IACUC-DWZX-027–20).

### Molecular cloning and bioinformatics of *HlHSP20*

The *HlHSP20* gene sequence was initially predicted from RNA-sequencing (RNA-seq) data (GenBank accession: SRR30735266-SRR30735286) [12]. Total RNA was extracted from engorged adult ticks using TRIzol reagent. cDNA was synthesized using the CloneMiner™ II cDNA Library Construction Kit (Invitrogen, USA). The reaction conditions were as follows: 65 °C for 5 min; ice bath for 2 min; 42 °C for 15 min. The full-length HlHSP20 gene was amplified using PrimeSTAR HS DNA Polymerase (Takara Bio, China) with specific primers (HSP-F: 5-ATGGCGCCCGAACGCCGC GT-3; HSP-R: 5-CTACTTCTTGTCGATGGGGATG TTGC-3). The PCR conditions were: 95 °C for 5 min; 45 cycles of 95 °C for 40 s, 55 °C for 30 s, 72 °C for 2 min; final extension at 72 °C for 10 min. The PCR products were purified using a Gel Extraction Kit (QIAGEN, Germany), ligated into the pGEX-6p-1 expression vector. The ligated product was transformed into *Escherichia coli* DH10B competent cells for prokaryotic expression. Positive clones were selected and cultured, and plasmids were extracted. Sequencing was performed using universal M13F/R primers at BGI (Beijing Genomics Institute, China). The obtained gene sequence was verified by plasmid sequencing and subsequently subjected to comparative alignment with the *HlHSP20* gene mRNA sequence from transcriptomic data.

Predicted physicochemical properties of HlHSP20 were analyzed by ProtParam (https://web.expasy.org/protparam/). Secondary structure was predicted using SOPMA (https://npsa-pbil.ibcp.fr/cgi-bin/npsa_automat.pl?page=npsa_sopma.html). Transmembrane domains were analyzed using TMHMM 2.0 (http://www.cbs.dtu.dk/ services/ TMHMM/). The tertiary structure of HlHSP20 was predicted using the SWISS-MODEL server (https://swissmodel.expasy.org/). Homologous amino acid sequences of HlHSP20 were aligned using MAFFT 7.0. A phylogenetic tree was built with IQ-TREE software (http://www.iqtree.org/) using the maximum likelihood method (1,000 bootstrap replicates) on the basis of sequences from various hard tick species (Ixodidae).

### Expression dynamics of *HlHSP20* across developmental stages

*HlHSP20* expression was quantified across developmental stages: eggs (10 days post-oviposition), unfed larvae, unfed nymphs, and unfed adults (14 days post-molting) from parthenogenetic *H. longicornis*. Quantitative real-time PCR (qPCR) was performed using primers designed with Primer Premier 5 and validated by Sanger sequencing (qPCR-F: 5-CAAGATGGAGGAGGAGATGT-3; qPCR-R: 5-CGAGTTCATGCTGTC CAG-3). cDNA was synthesized using Takara CN830A Real-Time PCR Kit (TaKaRa Bio, China). qPCR reactions (10 µl) were conducted with Takara SYBR Green qPCR Master Mix. The reaction conditions were as follows: pre-denaturation: 94 °C for 30 s; PCR reaction: 40 cycles of 94 °C for 5 s and 60 °C for 30 s; hold: 65 °C for 5 s and 95 °C for 5 s. Unfed adult ticks served as the control group. Relative gene expression was quantified using the 2^−ΔΔCt^ method. Statistical analysis was performed using GraphPad Prism 8.0 (Student’s *t*-test, *P < 0.05, **P < 0.01, ***P < 0.001).

### RNA interference-mediated knockdown of *HlHSP20* and *R. heilongjiangensis* proliferation assay

Double-stranded RNA (dsRNA) targeting *HlHSP20* was synthesized using the Promega T7 RiboMAX™ Express RNAi System, with primers incorporating T7 promoter sequences (RNAi-F: 5-ACCACGAGCGAGACTTCTTC-3; RNAi-R: 5-GACTTCTC CTCATGCTTGGC-3). dsRNA concentration and integrity were assessed by NanoDrop 2000 (Thermo Fisher Scientific, USA) and gel electrophoresis (QIAGEN, Germany), adjusted to 5 µg/µl. GFP-targeting dsRNA served as a negative control. Unfed adult ticks were microinjected with 2 µl of dsRNA (10 µg total) into the ventral hemocoel. RNAi efficiency was evaluated by qPCR at 72 h. Ticks were then microinjected with *R. heilongjiangensis*. Pathogen load was quantified at 5, 10, 15, and 20 days post infection (dpi) by quantifying the genomic copy number of the *R. heilongjiangensis* Sca1 gene using qPCR. Total DNA was extracted from individual ticks, and the pathogen load was calculated relative to a standard curve of *R. heilongjiangensis* genomic DNA using specific primers (R.HLJ-F: 5-GTTTGTGGATG CGTGGTA-3; R.HLJ-R: 5-AACCCGATAGTAGCAC-3).

### Identification of HlHSP20-interacting proteins

A GST-tagged recombinant HlHSP20 protein was expressed in *E. coli* using pGEX-6p-1 and purified via GST affinity chromatography. Protein purity was confirmed by sodium dodecyl sulfate–polyacrylamide gel electrophoresis (SDS–PAGE). For GST pulldown assays, immobilized GST-HlHSP20 was incubated with total protein lysate from *R. heilongjiangensis* cultured in Vero cells. A GST-Tag control assessed nonspecific binding. Bound proteins were eluted and analyzed by silver staining. Specific bands were excised and subjected to data-independent acquisition (DIA) analysis on a liquid chromatography–tandem mass spectrometry (LC–MS/MS) platform for quantitative proteomics to identify differentially expressed proteins (DEPs). We used AlphaFold3 (https://alphafoldserver.com/), ClusPro (https://cluspro.org/), and DMFold computer projections for DEPs (https://zhanggroup.org/DMFold/) to determine the candidate protein interaction with HlHSP20.

Three *R. heilongjiangensis* proteins with strong predicted interaction potential with HlHSP20 were selected for yeast two-hybrid (Y2H) experiment. The pGBKT7-HSP20 bait vector and the genes for the three selected *R. heilongjiangensis* proteins (cloned into pGADT7 prey vector) were co-transformed into Y2HGold yeast. Transformants were plated on stringent selective media: SD/-Leu/-Trp (DDO/X), followed by SD/-Leu/-Trp/-His/X-α-gal (TDO/X), and finally SD/-Leu/-Trp/-His/-Ade/X-α-gal/AbA (QDO/X/A). Standard positive (pGBKT7-53 + pGADT7-T) and negative (pGBKT7-Lam + pGADT7-T) controls, along with a bait auto-activation control (pGBKT7-HSP20 + empty pGADT7), were included. The growth on the QDO/X/A medium and the blue colony formation confirmed specific interactions between HSP20 and the selected *R. heilongjiangensis* proteins.

## Results

### Molecular characterization and sequence validation of *HlHSP20*

**Fig. 1:**
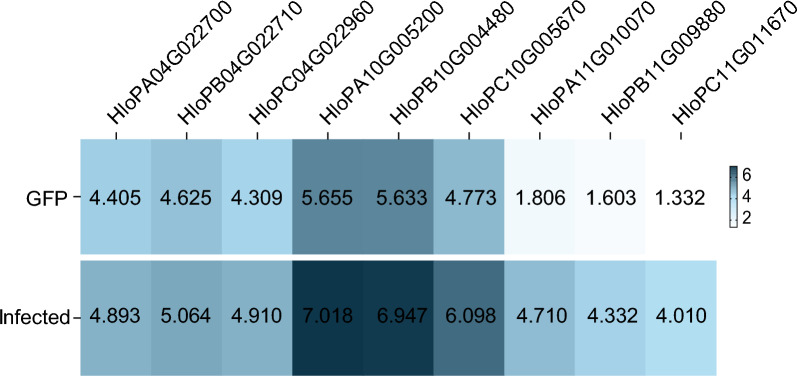
Transcriptome expression analysis of *HSP* family genes in *H. longicornis* after *R. heilongjiangensis* infection. Transcript expression levels are presented as log₂-transformed TPM values. The color scale from white to blue indicates increasing transcript expression levels (white: low expression; blue: high expression). The control groups represent uninfected ticks, and the experimental groups represent infected ticks. Each group consisted of three biological replicates. Differences between control and infected groups were analyzed by the Wilcoxon rank-sum test. *P* < 0.05 was considered statistically significant. Genes include *HSP20* (HloPA04G022700, HloPB04G022710, HloPC04G022960), *HSP70* (HloPA10G005200, HloPB10G004480, HloPC10G005670), and *HSP90* (HloPA11G010070, HloPB11G009880, HloPC11G011670)

In total, nine *HSPs* transcripts (including *HSP20*, *HSP70*, *HSP90*) were identified by transcriptomic analysis of *R. heilongjiangensis*-infected adult ticks, using the available genome [12]. One transcript (GenBank project: Gene ID: HloPC04G022960) exhibited significant upregulation (Wilcoxon rank-sum test, *P < 0.05) in infected ticks compared with uninfected controls (Fig. 1). This gene was cloned, and full-length sequencing confirmed the *HlHSP20* gene sequence, which was consistent with the transcriptome data. The deduced amino acid sequence of HlHSP20 is provided in Supplementary Fig. S1a. Bioinformatics analysis predicted that the HlHSP20 protein contained 187 amino acids with a molecular weight of 21.5 kDa. Its theoretical isoelectric point (pI) was 5.76, aliphatic index was 71.98, instability index was 62.61, and GRAVY score was −0.853, classifying it as a hydrophobic protein. Secondary structure prediction via SOPMA revealed a composition of 49.20% random coil, 28.88% α-helix, and 5.35% β-sheet (Table 1).

**Table 1:** Physicochemical properties and secondary structure characterization of HlHSP20

Protein	Physicochemical property						Proportion of secondary structures		
	Amino acid number	Molecular weight (Da)	Theoretical isoelectric point	Instability index	Aliphatic index	GRAVY	Alpha helix (Hh)	Extended strand (Ee)	Random coil (Cc)
*HlHSP20*	187	21456.28	5.76	62.61	71.98	−0.853	28.88%	16.58%	49.20%

### Molecular properties and phylogenetic analysis of *HlHSP20*

**Fig. 2:**
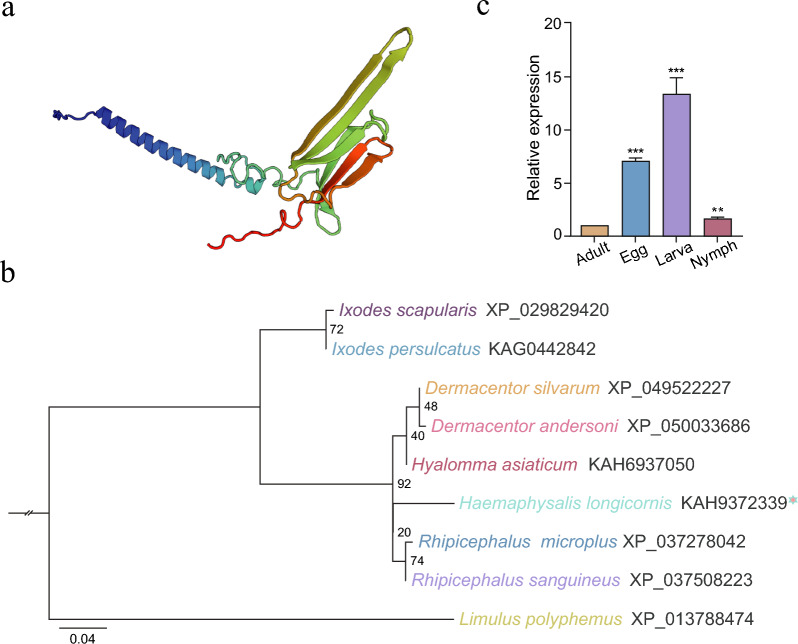
Molecular characterization and developmental expression profile of HlHSP20. **a** Tertiary structure prediction of HlHSP20. The model, generated by SWISS-MODEL; global model quality estimation (GMQE) score of 76%. **b** maximum likelihood (ML) phylogenetic tree of tick *HSP20* genes. The tree was constructed using IQ-TREE with 1,000 bootstrap replicates. **c** Developmental stage-specific expression of *HSP20.* The qPCR was used to measure *HSP20* transcript levels in eggs (10 days post-oviposition), larvae, nymphs, and adult ticks (2–4 weeks post-molting). Data are presented as mean ± standard deviation (SD) (*n* = 3). Statistical significance was determined by Student’s *t*-test compared with the adult group (control), with egg stage *t*(4) = 24.84, larval stage *t*(4) = 30.95, nymphal stage *t*(4) = 7.135; **P < 0.01 and ***P < 0.001

TMHMM 2.0 analysis revealed an ExpAA value of 0.00023, a first-60 value of 0.00008, and an N-in probability of 0.14612, indicating that HlHSP20 lacks transmembrane domains and can be classified as a non-transmembrane protein (Supplementary Fig. S1b). The tertiary structure prediction using Swiss-model showed 70.11% sequence similarity to the alpha-crystallin B chain isoform X protein (AlphaFold DB ID: A0A7M7R4W5.1.A) from *Apis mellifera*, placing it within the small heat-shock protein (sHSP) family as an αB-crystallin isoform of HlHSP20. The high GMQE score (76%) indicated a reliable model and strong conservation of the protein structure among tick species (Fig. 2a). Homologous sequence searches confirmed *HlHSP20* as a canonical sHSP gene in ticks (Supplementary Fig. S1c). Phylogenetic analysis using the maximum likelihood (ML) method in IQ-TREE software revealed that the *HlHSP20* gene clusters with sequences from *Rhipicephalus sanguineus* and *Rhipicephalus microplus* (bootstrap value = 92), demonstrating high genetic conservation within the *Rhipicephalus* genus and clear divergence from the *Ixodes* and *Dermacentor* genera, consistent with the classical tick taxonomic framework (Fig. 2b).

### Developmental stage-specific expression of *HlHSP20*

The qPCR was employed to analyze *HlHSP20* gene expression across different developmental stages of parthenogenetic *H. longicornis*. Significant differences in expression dynamics were observed among these stages relative to adults (Fig. 2c). Notably, *HlHSP20* expression was approximately 7.1-fold higher in eggs, peaked at 13.4-fold in larvae, and then decreased sharply to 1.65-fold in nymphs. The dynamic expression of *HlHSP20*, with high levels during egg and larval stages in *H. longicornis*, indicates that it likely plays an important role in early development by maintaining cellular homeostasis and adapting to environmental stress.

### RNA interference of *HlHSP20* affects *R. heilongjiangensis* proliferation

**Fig. 3:**
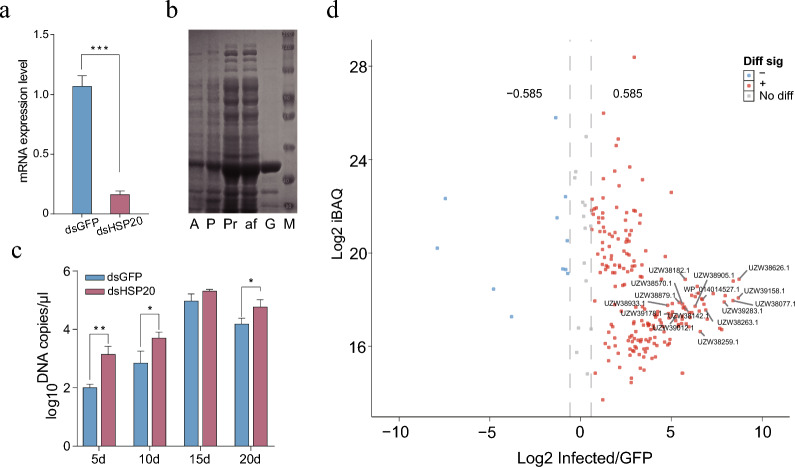
RNA interference, protein purification, and pathogen interaction analysis. **a** RNAi treatment result in an 85% reduction in *HlHSP20* expression at 72 h. Data are presented as mean ± standard deviation (SD) (*n* = 3, Student’s *t*-test, *t*(4) = 16.82), with ***P < 0.001 compared with the dsGFP group. **b** SDS–PAGE of purified GST-HlHSP20 fusion protein. Lanes: A (protein bands after induction), P (insoluble pellet), Pr (crude supernatant), af (flow-through liquid), G (eluted GST-*HlHSP20* fusion protein), M (molecular weight marker). **c** *R. heilongjiangensis* proliferation following *HlHSP20* silencing. Pathogen load was quantified by qPCR at 5–20 days post-infection (dpi). Data are presented as log10 DNA copies/μl (mean ± SD, *n* = 3, Student’s *t*-test), with 5 dpi *t*(4) = 5.600, 10 dpi *t*(4) = 2.875, 15 dpi *t*(4) = 2.046, 20 dpi *t*(4) = 2.926; *P < 0.05, **P < 0.01 compared with the dsGFP group. **d** Volcano plot of DEPs identified by LC–MS/MS. A total of 206 DEPs were identified in GST pulldown assays: 195 were upregulated (red dots), 11 were downregulated (blue dots)

To investigate the functional role of *HlHSP20* in the interaction between *H. longicornis* and *R. heilongjiangensis*, adult ticks were microinjected with either *HlHSP20*-specific dsRNA (dsHSP20 group) and GFP-targeting dsRNA (dsGFP group) as a negative control. The qPCR confirmed efficient knockdown of *HlHSP20* mRNA levels (85% reduction) in the dsHSP20 group at 72 h (Fig. 3a). Subsequently, ticks were reinfected with *R. heilongjiangensis*, and pathogen proliferation was assessed at 5, 10, 15, and 20 dpi. The *R. heilongjiangensis* DNA copy number (log10 DNA copies/μl) was consistently higher in the dsHSP20 group compared with the dsGFP group across all time points (Fig. 3c). These findings indicate that silencing *HlHSP20* disrupts *H. longicornis* physiological functions, thereby promoting pathogen replication and dissemination. This suggests *HlHSP20* plays a significant role in the tick’s resistance to rickettsial infection.

### Prokaryotic expression, purification, and interaction screening of *HlHSP20*

SDS–PAGE confirmed the successful induction and expression of a predominantly soluble HlHSP20 fusion protein, which was subsequently purified via GST affinity chromatography for interaction assays (Fig. 3b). A GST pulldown assay using recombinant GST-HlHSP20 against *R. heilongjiangensis* total protein lysate revealed specific protein interactions (Supplementary Fig. S2). Subsequent LC–MS/MS analysis of the excised protein bands revealed 206 differentially expressed proteins (DEPs), of which 195 were upregulated, and 11 were downregulated (Fig. 3d). The upregulation of the vast majority of interacting rickettsial proteins further suggests that HlHSP20 may be involved in cellular defense or response mechanisms aimed at countering or regulating pathogen activity (Supplementary Table S1).

On the basis of these proteomic findings and subsequent in silico protein–protein interaction prediction using AlphaFold3, Dmfold, and Cluspro, four potential interacting proteins (Genbank IDs: UZW39283.1, UZW38525.1, UZW38371.1, and UZW38497.1) were prioritized for further experimental validation (Table 2). This selection was based on consistently high interaction scores across multiple prediction tools, evidenced by strong Alphafold3 ipTM/pTM values (0.61–0.67 / 0.60–0.69), favorable Dmfold Δ*G* (all negative, ranging from −3.9 to −11.8), and high Cluspro Balanced scores (from 77 to 114). The convergence of these high scores across different methods provided strong evidence for their potential functional relevance.

**Table 2:** Predicted protein–protein interactions between HlHSP20 and *R. heilongjiangensis* proteins

Genbank ID	Alphafold3		Dmfold						Cluspro		
	ipTM	pTM	DMfoldΔ*G* (kcal mol^−1^)	alphafoldΔ*G* (kcal mol^−1^)	A^2^ (alphafold)	A^2^ (DMfold)	pTM-score (DMfold)	Balanced	Electrostatic favored	Hydrophobic favored	VdW + Elec
UZW39283.1	0.61	0.6	−8.6	−9.5	1,698.548	1,580.951	0.68	103	94	281	84
UZW38525.1	0.62	0.69	−3.9	−8.0	1,774.931	66.884	0.29	79	63	129	67
UZW38371.1	0.62	0.61	−7.7	−7.4	927.855	1,155.96	0.70	77	69	142	53
UZW38497.1	0.67	0.64	−11.8	−14.1	4,495.211	3,203.136	0.38	114	66	202	134

### Yeast two-hybrid validation of *HlHSP20*-protein interactions

**Fig. 4:**
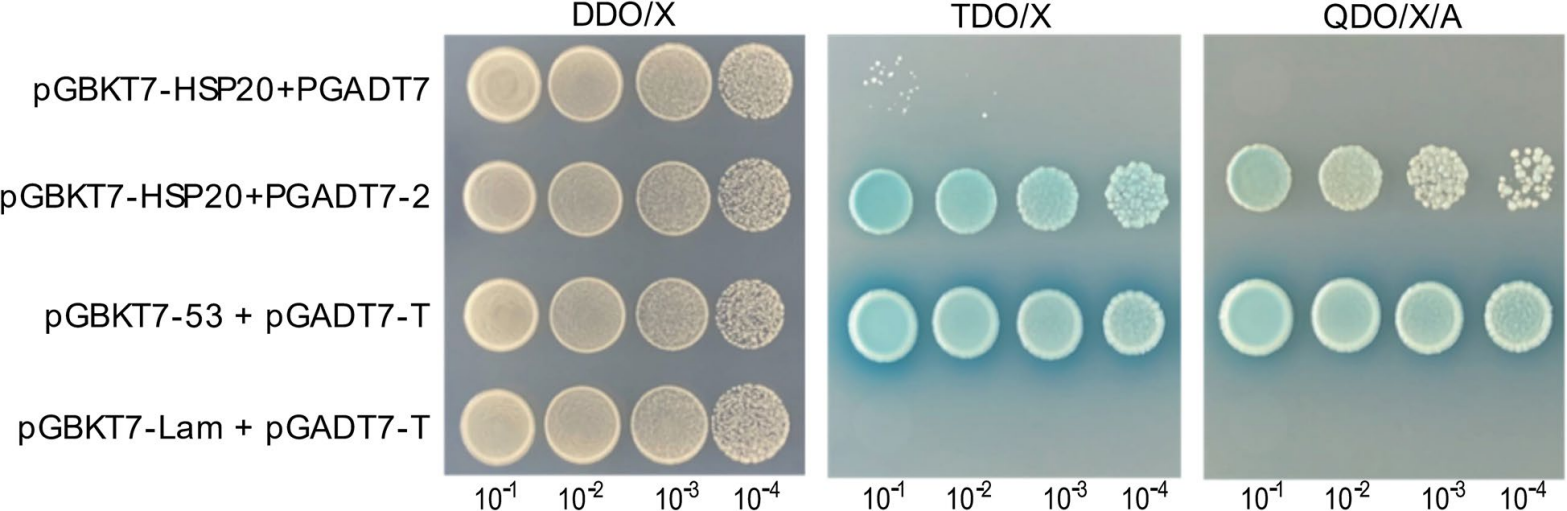
Yeast two-hybrid (Y2H) assay confirming the interaction between HlHSP20 and RhRPL14 protein. Yeast strains co-transformed with bait and prey vectors were serially diluted (10^−1^ to 10^−4^) and plated on different selective media to assess protein–protein interactions

Although four proteins showed potential interactions (Table 2), we prioritized the top three candidates with the highest consistent scores for Y2H validation, excluding UZW39283.1. The candidates were cloned into the pGADT7 prey vector: UZW38525.1 (construct: pGADT7-1), UZW38371.1 (pGADT7-2), and UZW38497.1 (pGADT7-3). Autoactivation assays confirmed the suitability of the Y2H system with no bait autoactivation (Supplementary Fig. S3a–c). The co-transforming pGBKT7-HSP20 with the candidate prey vectors indicated interaction exclusively with pGADT7-2 (UZW38371.1, 50S ribosomal protein L14, RhRPL14). Formal Y2H screening confirmed specific growth and blue coloration on selective media (TDO/X and QDO/X/A) for pGADT7-2, in contrast to pGADT7-1 and pGADT7-3, which showed no interaction signals (Fig. 4, Supplementary Fig. S3d–f). These results confirm a specific physical interaction between HlHSP20 and the RhRPL14 protein of *R. heilongjiangensis*. The lack of detectable interactions for the remaining candidates (UZW38525.1 and UZW38497.1) may be attributed to weak binding affinity or compromised protein folding and stability within the yeast system.

## Discussion

Here, we show that HlHSP20 is not only a molecular responder to cellular stress but also a host restriction factor that inhibits the replication of *R. heilongjiangensis*. Our findings reveal a direct interaction between this chaperone protein and the pathogen’s translation machinery, providing a new perspective on tick innate immunity. Notably, the RNAi-mediated knockdown of *HlHSP20* leads to a significant and sustained increase in pathogen load. This indicates that HlHSP20 is essential for ticks to control intracellular rickettsial infection, thereby redefining the functional scope of sHSPs in tick immunity. Although the roles of sHSPs in maintaining cellular homeostasis [13–15] are well-recognized, our research demonstrates that their function extends to active pathogen defense. The infection-induced upregulation of *HlHSP20* at the transcriptional level suggests a specific immune-related function rather than a broad stress response [16]. This finding supports an increasingly clear concept in arthropod immunology: that chaperones play a dual role in maintaining protein homeostasis and executing immune defense functions [17].

The identification of *HlHSP20* as a host restriction factor provides a new reference for chaperone-mediated immune modulation in arthropods. In arthropods lacking adaptive immunity, sHSPs serve as core effectors, playing crucial roles by directly targeting pathogen components or activating innate immune signals [9]. Studies on *Ixodes ricinus* have confirmed that the differential expression of the HSP family is correlated with the host’s pathogen resistance [11], providing background support for the significant induction of *HlHSP20* by rickettsial infection observed in this study. However, academic debates persist regarding the regulatory functions of these chaperones in host–pathogen interactions: while they act as defense sentinels to inhibit pathogen replication [17], they may also be hijacked by pathogens to facilitate assembly or invasion [18]. Our data demonstrate the specific mobilization of HlHSP20 during infection, supporting the model of its role as a targeted immune regulator.

To further elucidate the molecular basis of this modulation, we prioritized the investigation of the interaction between HlHSP20 and the rickettsial RhRPL14 protein. Among the rickettsial interacting proteins screened via GST pulldown and LC–MS/MS, RhRPL14 was identified as the top candidate across multiple protein–protein interaction prediction platforms. RhRPL14 is a core component of the bacterial 50S ribosomal subunit and a critical site for ribosome assembly and mRNA binding [19, 20]. The sequestration of RhRPL14 by HlHSP20 likely leads to severe translational dysfunction. This strategy of interfering with core pathogen metabolic pathways resembles the mode of action of antimicrobial peptides and conventional antibiotics, suggesting a sophisticated intracellular pathogen control mechanism for the host HlHSP20 protein. This discovery not only deepens the understanding of the innate immune mechanisms in ticks mediated by small heat shock proteins but also provides potential intervention targets for subsequently blocking the transmission of tick-borne rickettsial diseases.

## Conclusions

The present study demonstrates that HlHSP20 functions as a host restriction factor that contributes to limits the replication of *R. heilongjiangensis* in *H. longicornis*. While these findings reveal a potentially valuable chaperone-mediated host defense strategy, further structural studies and in vivo validations are required to fully elucidate the underlying biochemical mechanisms and the overall impact of this interaction on tick vector competence. Nevertheless, these findings identify a novel role for sHSPs in tick immunity and expand the current understanding of chaperone-mediated host defense against intracellular rickettsiae.

## Supplementary Information

**Additional file 1:**Fig. S1. Sequence and structural characterization of HlHSP20. Fig. S2. Silver staining analysis of proteins enriched by GST pull-down assays. Fig. S3. Validation of protein-protein interaction detection using the Y2H system. Table S1. Identification and differential expression analysis of proteins enriched by pull-down assays usingpurifiedR. *heilongjiangensis*proteins.

## Data Availability

The *Rickettsia heilongjiangensis* strain used in this study has been deposited in the NCBI database under GenBank accession number GCF_035117685. The animal ethics approval number is IACUC-DWZX-027-20. The RNA-seq data of uninfected ticks and ticks infected with *R. heilongjiangensis* at GenBank with accession numbers SRR30735266–SRR30735286. The GenBank accession number for the identified HlHSP20 gene transcript (Gene ID: HloPC04G022960) is PRJNA1133666. The numerical source data for all figures have been deposited in Figshare (10.6084/m9.figshare.30993535).
