# Supplementary material for: Haemaphysalis longicornis HSP20 inhibits Rickettsia heilongjiangensis replication by targeting the pathogen 50S ribosomal protein
Source: Parasit Vectors. 2026 Apr 8;19:217. doi: 10.1186/s13071-026-07398-x (PMC13185263; doi:10.1186/s13071-026-07398-x)
Supplement: Supplementary file 1 — Additional file 1:Fig. S1. Sequence and structural characterization of HlHSP20. Fig. S2. Silver staining analysis of proteins enriched by GST pull-down assays. Fig. S3. Validation of protein-protein interaction detection using the Y2H system. Table S1. Identification and differential expression analysis of proteins enriched by pull-down assays usingpurifiedR. heilongjiangensisproteins. [file 13071_2026_7398_MOESM1_ESM.docx]

**Supplementary Information**


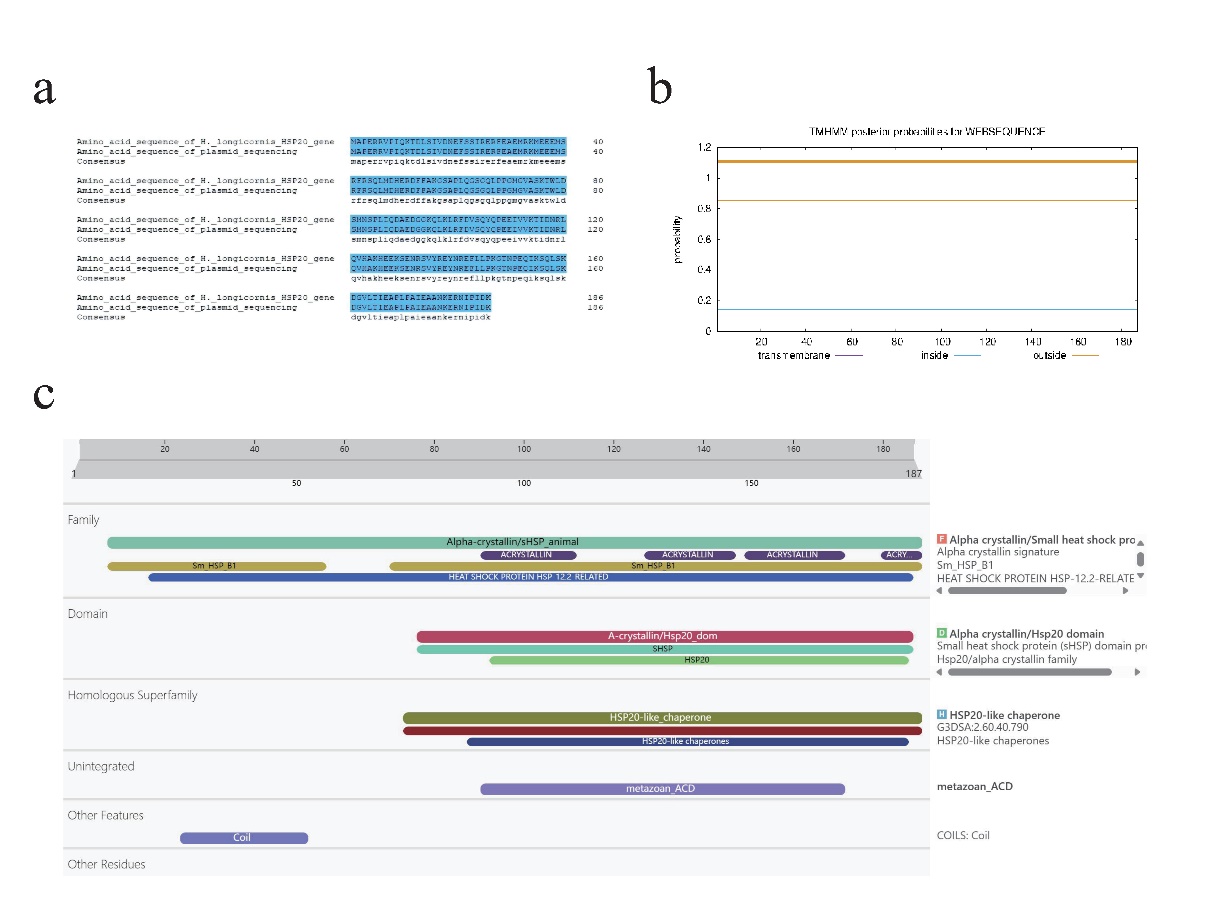


**Fig. S1 Sequence and structural characterization of HlHSP20. a** Deduced amino acid sequence of HlHSP20. The sequence was derived from the cloned *HlHSP20* gene (Gene ID: HloPC04G022960) and validated by Sanger sequencing. **b** Transmembrane domain prediction: Analysis using TMHMM 2.0 indicated an expected number of amino acids in transmembrane helices (ExpAA) of 0.00023, confirming HlHSP20 as a non-transmembrane protein. **c** Homologous sequence alignment: BLAST analysis revealed significant alignment of HlHSP20 with sHSPs from other tick species, verifying its canonical *sHSP* family membership.


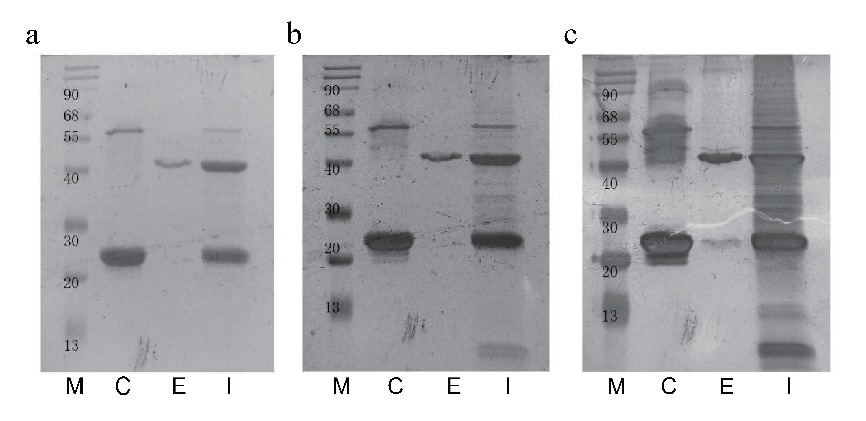


**Fig. S2 Silver staining analysis of proteins enriched by GST pull-down assays.** Protein molecular weight marker is indicated by (M. Differential protein bands were observed between the experimental group (E: GST-HlHSP20 beads incubated with *R. heilongjiangensis* lysate) and the control group (C: GST beads incubated with *R. heilongjiangensis* lysate) after incubation for 15 min (a), 30 min (b), and 16 h (c). INPUT (I) represents the total *R. heilongjiangensis* protein lysate.


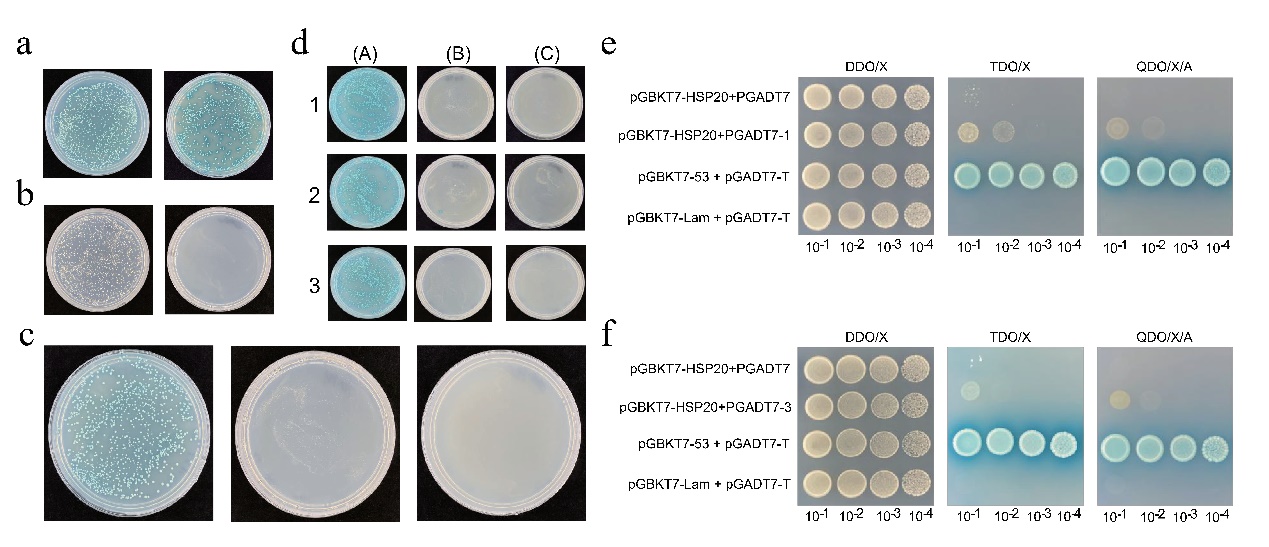


**Fig. S3 Validation of protein-protein interaction detection using the Y2H system. a** Positive control: Co-transformation of yeast cells with pGBKT7-53 and pGADT7-T resulted in growth and blue colony formation on both DDO/X (SD/-Leu/-Trp/X-α-gal) and QDO/X/A (SD/-Leu/-Trp/-His/-Ade/X-α-gal/AbA) plates, validating the Y2H system for PPI detection. **b** Negative control: Co-transformation with pGBKT7-Lam and pGADT7-T resulted in growth without blue coloration on DDO/X plates, and no growth on QDO/X/A plates, confirming the system’s specificity and absence of non-specific interactions. **c** Bait vector autoactivation assay: Co-transformation of pGBKT7-HSP20 with an empty pGADT7 vector led to growth without blue coloration on DDO/X plates and no growth on QDO/X/A plates, indicating no autoactivation of the bait vector. **d** Preliminary co-transformation assay: The recombinant bait vector (pGBKT7-HSP20) was co-transformed with prey vectors (pGADT7-1/2/3) or control plasmids into Y2HGold yeast cells. Growth and coloration were observed on DDO/X (A), TDO/X (SD/-Leu/-Trp/-His/X-α-gal; B), and QDO/X/A (C) plates. Lanes indicate: 1: pGBKT7-HSP20 + pGADT7-1; 2: pGBKT7-HSP20 + pGADT7-2; 3: pGBKT7-HSP20 + pGADT7-3; A: positive control (pGBKT7-53 + pGADT7-T); B: negative control (pGBKT7-Lam + pGADT7-T); C: empty vector control (pGBKT7-HSP20 + empty pGADT7). **e** Co-transformation of pGBKT7-HSP20 + pGADT7-1: Faint, non-blue colonies grew on TDO/X plates, with no growth on QDO/X/A plates. **f** Co-transformation of pGBKT7-HSP20 + empty pGADT7 / pGADT7-3: Yeast cells grew without blue coloration on DDO/X plates, and showed no growth on TDO/X or QDO/X/A plates.

**Table S1 Identification and differential expression analysis of proteins enriched by pull-down assays using purified *R. heilongjiangensis* proteins.** Proteins were identified and quantified by mass spectrometry following pull-down assays. Log_2_ Fold Change (Log_2_ FC) represents the log₂-transformed ratio between protein enrichment levels in the *R. heilongjiangensis* protein group and the GST control group. Symbols indicate differential expression: ‘+’ denotes significant upregulation, ‘-’ denotes significant downregulation, and blank entries indicate no significant change.

| **Genbank accession** | **NR Annotation description** | **Significant difference** | **Log_2_ FC** |
| --- | --- | --- | --- |
| UZW38626.1 | single-stranded DNA-binding protein [*Rickettsia conorii* subsp. *heilongjiangensis*] | + | 8.720 |
| UZW39158.1 | enoyl-ACP reductase FabI [*Rickettsia conorii* subsp. *heilongjiangensis*] | + | 8.687 |
| UZW38312.1 | 50S ribosomal protein L34 [*Rickettsia conorii* subsp. *heilongjiangensis*] | + | 8.401 |
| UZW38077.1 | ribonucleotide-diphosphate reductase subunit beta [*Rickettsia conorii* subsp. *heilongjiangensis*] | + | 8.386 |
| BBM95515.1 | acyl carrier protein [*Rickettsia conorii* subsp. *heilongjiangensis*] | + | 7.954 |
| UZW39283.1 | ribosome-associated translation inhibitor RaiA [*Rickettsia conorii* subsp. *heilongjiangensis*] | + | 7.904 |
| UZW38525.1 | 3-oxoacyl-ACP reductase FabG [*Rickettsia conorii* subsp. *heilongjiangensis*] | + | 7.766 |
| UZW38370.1 | 50S ribosomal protein L24 [*Rickettsia conorii* subsp. *heilongjiangensis*] | + | 7.662 |
| UZW38363.1 | 50S ribosomal protein L30 [*Rickettsia conorii* subsp. *heilongjiangensis*] | + | 7.296 |
| UZW38981.1 | 30S ribosomal protein S9 [*Rickettsia conorii* subsp. *heilongjiangensis*] | + | 6.965 |
| UZW38263.1 | UDP-N-acetylglucosamine 1-carboxyvinyltransferase [*Rickettsia conorii* subsp. *heilongjiangensis*] | + | 6.887 |
| UZW38810.1 | pyridoxal phosphate-dependent aminotransferase [*Rickettsia conorii* subsp. *heilongjiangensis*] | + | 6.791 |
| WP_014014527.1 | peptidylprolyl isomerase [*Rickettsia conorii*] | + | 6.701 |
| UZW38598.1 | translation initiation factor IF-1 [*Rickettsia conorii* subsp. *heilongjiangensis*] | + | 6.661 |
| UZW38259.1 | preprotein translocase subunit SecA [*Rickettsia conorii* subsp. *heilongjiangensis*] | + | 6.590 |
| UZW39372.1 | hypothetical protein OSR38_00415 [*Rickettsia conorii* subsp. *heilongjiangensis*] | + | 6.534 |
| UZW38838.1 | DUF2312 domain-containing protein [*Rickettsia conorii* subsp. *heilongjiangensis*] | + | 6.480 |
| WP_014013989.1 | MULTISPECIES: DUF2671 domain-containing protein [spotted fever group] | + | 6.416 |
| UZW38372.1 | 30S ribosomal protein S17 [*Rickettsia conorii* subsp. *heilongjiangensis*] | + | 6.310 |
| UZW38905.1 | 2-oxoglutarate dehydrogenase complex dihydrolipoyllysine-residue succinyltransferase [*Rickettsia conorii* subsp. *heilongjiangensis*] | + | 6.301 |
| UZW39249.1 | succinate--CoA ligase subunit alpha [*Rickettsia conorii* subsp. *heilongjiangensis*] | + | 6.125 |
| UZW38645.1 | glycine--tRNA ligase subunit alpha [*Rickettsia conorii* subsp. *heilongjiangensis*] | + | 6.107 |
| UZW38987.1 | elongation factor P [*Rickettsia conorii* subsp. *heilongjiangensis*] | + | 6.025 |
| UZW38754.1 | 30S ribosomal protein S18 [*Rickettsia conorii* subsp. *heilongjiangensis*] | + | 5.971 |
| UZW38364.1 | 30S ribosomal protein S5 [*Rickettsia conorii* subsp. *heilongjiangensis*] | + | 5.966 |
| UZW38870.1 | aspartate--tRNA ligase [*Rickettsia conorii* subsp. *heilongjiangensis*] | + | 5.810 |
| UZW38454.1 | hypothetical protein OSR38_05740 [*Rickettsia conorii* subsp. *heilongjiangensis*] | + | 5.790 |
| UZW38182.1 | porphobilinogen synthase [*Rickettsia conorii* subsp. *heilongjiangensis*] | + | 5.763 |
| UZW38142.1 | folate-binding protein YgfZ [*Rickettsia conorii* subsp. *heilongjiangensis*] | + | 5.720 |
| UZW38376.1 | 50S ribosomal protein L22 [*Rickettsia conorii* subsp. *heilongjiangensis*] | + | 5.659 |
| UZW38871.1 | hypothetical protein OSR38_01010 [*Rickettsia conorii* subsp. *heilongjiangensis*] | + | 5.609 |
| UZW38570.1 | aconitate hydratase AcnA [*Rickettsia conorii* subsp. *heilongjiangensis*] | + | 5.609 |
| UZW39229.1 | diaminopimelate epimerase [*Rickettsia conorii* subsp. *heilongjiangensis*] | + | 5.600 |
| UZW38587.1 | ribosome silencing factor [*Rickettsia conorii* subsp. *heilongjiangensis*] | + | 5.597 |
| UZW38726.1 | class I SAM-dependent methyltransferase [*Rickettsia conorii* subsp. *heilongjiangensis*] | + | 5.571 |
| UZW38879.1 | ribosome recycling factor [*Rickettsia conorii* subsp. *heilongjiangensis*] | + | 5.438 |
| BBM95109.1 | peptide chain release factor 1 [*Rickettsia conorii* subsp. *heilongjiangensis*] | + | 5.395 |
| UZW38527.1 | beta-ketoacyl-ACP synthase II [*Rickettsia conorii* subsp. *heilongjiangensis*] | + | 5.391 |
| UZW39012.1 | pyruvate dehydrogenase complex E1 component subunit beta [*Rickettsia conorii* subsp. *heilongjiangensis*] | + | 5.262 |
| WP_267185284.1 | DUF2659 family protein [*Rickettsia conorii*] | + | 5.247 |
| UZW38379.1 | 50S ribosomal protein L23 [*Rickettsia conorii* subsp. *heilongjiangensis*] | + | 5.064 |
| UZW39185.1 | proline--tRNA ligase [*Rickettsia conorii* subsp. *heilongjiangensis*] | + | 5.060 |
| UZW38753.1 | 30S ribosomal protein S6 [*Rickettsia conorii* subsp. *heilongjiangensis*] | + | 5.004 |
| BBM94754.1 | hypothetical protein RHSENDAI58_01670 [*Rickettsia conorii* subsp. *heilongjiangensis*] | + | 5.001 |
| UZW38205.1 | tyrosine--tRNA ligase [*Rickettsia conorii* subsp. *heilongjiangensis*] | + | 4.928 |
| WP_045805686.1 | MULTISPECIES: NAD(P) transhydrogenase subunit alpha [spotted fever group] | + | 4.894 |
| UZW38378.1 | 50S ribosomal protein L2 [*Rickettsia conorii* subsp. *heilongjiangensis*] | + | 4.875 |
| UZW39178.1 | malate dehydrogenase [*Rickettsia conorii* subsp. *heilongjiangensis*] | + | 4.844 |
| UZW38381.1 | 50S ribosomal protein L3 [*Rickettsia conorii* subsp. *heilongjiangensis*] | + | 4.808 |
| UZW38933.1 | TAXI family TRAP transporter solute-binding subunit [*Rickettsia conorii* subsp. *heilongjiangensis*] | + | 4.797 |
| UZW38524.1 | recombinase RecA [*Rickettsia conorii* subsp. *heilongjiangensis*] | + | 4.675 |
| UZW38863.1 | DNA-directed RNA polymerase subunit beta [*Rickettsia conorii* subsp. *heilongjiangensis*] | + | 4.631 |
| UZW38575.1 | dihydrolipoyl dehydrogenase [*Rickettsia conorii* subsp. *heilongjiangensis*] | + | 4.600 |
| UZW38329.1 | acetyl/propionyl/methylcrotonyl-CoA carboxylase subunit alpha [*Rickettsia conorii* subsp. *heilongjiangensis*] | + | 4.546 |
| UZW38357.1 | DNA-directed RNA polymerase subunit alpha [*Rickettsia conorii* subsp. *heilongjiangensis*] | + | 4.459 |
| UZW39122.1 | UDP-N-acetylglucosamine 2-epimerase (non-hydrolyzing) [*Rickettsia conorii* subsp. *heilongjiangensis*] | + | 4.453 |
| UZW38366.1 | 50S ribosomal protein L6 [*Rickettsia conorii* subsp. *heilongjiangensis*] | + | 4.377 |
| UZW38980.1 | 50S ribosomal protein L13 [*Rickettsia conorii* subsp. *heilongjiangensis*] | + | 4.253 |
| UZW38371.1 | 50S ribosomal protein L14 [*Rickettsia conorii* subsp. *heilongjiangensis*] | + | 4.232 |
| UZW39296.1 | ribonucleoside-diphosphate reductase subunit alpha [*Rickettsia conorii* subsp. *heilongjiangensis*] | + | 4.209 |
| UZW38694.1 | protoporphyrinogen oxidase HemJ [*Rickettsia conorii* subsp. *heilongjiangensis*] | + | 4.140 |
| UZW38992.1 | HlyD family secretion protein [*Rickettsia conorii* subsp. *heilongjiangensis*] | + | 4.129 |
| UZW38434.1 | 7-cyano-7-deazaguanine synthase QueC [*Rickettsia conorii* subsp. *heilongjiangensis*] | + | 4.116 |
| UZW39082.1 | protein TolR [*Rickettsia conorii* subsp. *heilongjiangensis*] | + | 4.094 |
| UZW38265.1 | TIGR01459 family HAD-type hydrolase [*Rickettsia conorii* subsp. *heilongjiangensis*] | + | 4.059 |
| UZW38766.1 | hypothetical protein OSR38_00420 [*Rickettsia conorii* subsp. *heilongjiangensis*] | + | 4.052 |
| UZW39327.1 | ATP-dependent Clp endopeptidase proteolytic subunit ClpP [*Rickettsia conorii* subsp. *heilongjiangensis*] | + | 4.013 |
| WP_014014848.1 | MULTISPECIES: SurA N-terminal domain-containing protein [spotted fever group] | + | 3.937 |
| WP_041404383.1 | MULTISPECIES: HIT domain-containing protein [spotted fever group] | + | 3.878 |
| UZW39089.1 | aspartate-semialdehyde dehydrogenase [*Rickettsia conorii* subsp. *heilongjiangensis*] | + | 3.836 |
| WP_014014389.1 | MULTISPECIES: NTP transferase domain-containing protein [spotted fever group] | + | 3.776 |
| UZW39264.1 | ribonuclease J [*Rickettsia conorii* subsp. *heilongjiangensis*] | + | 3.761 |
| UZW39239.1 | ribonuclease D [*Rickettsia conorii* subsp. *heilongjiangensis*] | + | 3.755 |
| UZW38965.1 | threonine--tRNA ligase [*Rickettsia conorii* subsp. *heilongjiangensis*] | + | 3.734 |
| UZW38374.1 | 50S ribosomal protein L16 [*Rickettsia conorii* subsp. *heilongjiangensis*] | + | 3.701 |
| UZW38081.1 | LPS export ABC transporter ATP-binding protein [*Rickettsia conorii* subsp. *heilongjiangensis*] | + | 3.667 |
| UZW39066.1 | ribose 5-phosphate isomerase B [*Rickettsia conorii* subsp. *heilongjiangensis*] | + | 3.605 |
| UZW38787.1 | arginine--tRNA ligase [*Rickettsia conorii* subsp. *heilongjiangensis*] | + | 3.593 |
| WP_014014162.1 | DUF2608 domain-containing protein [*Rickettsia conorii*] | + | 3.578 |
| UZW39168.1 | lysine--tRNA ligase [*Rickettsia conorii* subsp. *heilongjiangensis*] | + | 3.534 |
| UZW38864.1 | DNA-directed RNA polymerase subunit beta’ [*Rickettsia conorii* subsp. *heilongjiangensis*] | + | 3.499 |
| UZW38839.1 | protein translocase subunit SecF [*Rickettsia conorii* subsp. *heilongjiangensis*] | + | 3.490 |
| UZW39115.1 | paraslipin [*Rickettsia conorii* subsp. *heilongjiangensis*] | + | 3.483 |
| UZW38476.1 | ACP S-malonyltransferase [*Rickettsia conorii* subsp. *heilongjiangensis*] | + | 3.471 |
| UZW39170.1 | hypothetical protein OSR38_02745 [*Rickettsia conorii* subsp. *heilongjiangensis*] | + | 3.414 |
| UZW38392.1 | cold-shock protein [*Rickettsia conorii* subsp. *heilongjiangensis*] | + | 3.412 |
| UZW38804.1 | 30S ribosomal protein S2 [*Rickettsia conorii* subsp. *heilongjiangensis*] | + | 3.387 |
| UZW38086.1 | 30S ribosomal protein S15 [*Rickettsia conorii* subsp. *heilongjiangensis*] | + | 3.385 |
| UZW39278.1 | hypothetical protein OSR38_03360 [*Rickettsia conorii* subsp. *heilongjiangensis*] | + | 3.378 |
| UZW39315.1 | NADP-dependent malic enzyme [*Rickettsia conorii* subsp. *heilongjiangensis*] | + | 3.345 |
| UZW38337.1 | invasion associated locus B family protein [*Rickettsia conorii* subsp. *heilongjiangensis*] | + | 3.305 |
| UZW38307.1 | 50S ribosomal protein L25/general stress protein Ctc [*Rickettsia conorii* subsp. *heilongjiangensis*] | + | 3.302 |
| UZW38633.1 | trigger factor [*Rickettsia conorii* subsp. *heilongjiangensis*] | + | 3.298 |
| UZW39205.1 | hypothetical protein OSR38_02955 [*Rickettsia conorii* subsp. *heilongjiangensis*] | + | 3.295 |
| UZW39367.1 | thioredoxin [*Rickettsia conorii* subsp. *heilongjiangensis*] | + | 3.265 |
| UZW39030.1 | translation elongation factor 4 [*Rickettsia conorii* subsp. *heilongjiangensis*] | + | 3.233 |
| UZW39254.1 | S9 family peptidase [*Rickettsia conorii* subsp. *heilongjiangensis*] | + | 3.174 |
| UZW38303.1 | redox-regulated ATPase YchF [*Rickettsia conorii* subsp. *heilongjiangensis*] | + | 3.069 |
| UZW39134.1 | 30S ribosomal protein S4 [*Rickettsia conorii* subsp. *heilongjiangensis*] | + | 3.049 |
| UZW38964.1 | phosphoribosylaminoimidazolesuccinocarboxamide synthase [*Rickettsia conorii* subsp. *heilongjiangensis*] | + | 3.045 |
| UZW38121.1 | 30S ribosomal protein S1 [*Rickettsia conorii* subsp. *heilongjiangensis*] | + | 3.019 |
| UZW38748.1 | ATP-dependent chaperone ClpB [*Rickettsia conorii* subsp. *heilongjiangensis*] | + | 3.003 |
| WUV41372.1 | surface cell antigen 4 [*Rickettsia conorii* subsp. *heilongjiangensis*] | + | 2.988 |
| WP_323811262.1 | autotransporter outer membrane beta-barrel domain-containing protein, partial [*Rickettsia conorii*] | + | 2.969 |
| Target | - | + | 2.962 |
| UZW38886.1 | outer membrane protein assembly factor BamA [*Rickettsia conorii* subsp. *heilongjiangensis*] | + | 2.947 |
| UZW38815.1 | phospholipid-binding protein MlaC [*Rickettsia conorii* subsp. *heilongjiangensis*] | + | 2.945 |
| UZW39087.1 | HlyD family type I secretion periplasmic adaptor subunit [*Rickettsia conorii* subsp. *heilongjiangensis*] | + | 2.936 |
| UZW39233.1 | DNA polymerase III subunit beta [*Rickettsia conorii* subsp. *heilongjiangensis*] | + | 2.935 |
| UZW38643.1 | L-threonylcarbamoyladenylate synthase [*Rickettsia conorii* subsp. *heilongjiangensis*] | + | 2.921 |
| UZW38962.1 | pitrilysin family protein [*Rickettsia conorii* subsp. *heilongjiangensis*] | + | 2.875 |
| UZW38644.1 | glycine--tRNA ligase subunit beta [*Rickettsia conorii* subsp. *heilongjiangensis*] | + | 2.853 |
| UZW38100.1 | superoxide dismutase [*Rickettsia conorii* subsp. *heilongjiangensis*] | + | 2.850 |
| UZW39016.1 | OmpH family outer membrane protein [*Rickettsia conorii* subsp. *heilongjiangensis*] | + | 2.823 |
| UZW38631.1 | molecular chaperone HtpG [*Rickettsia conorii* subsp. *heilongjiangensis*] | + | 2.814 |
| UZW38906.1 | 2-oxoglutarate dehydrogenase E1 component [*Rickettsia conorii* subsp. *heilongjiangensis*] | + | 2.805 |
| BBM94771.1 | phosphatidylserine decarboxylase [*Rickettsia conorii* subsp. *heilongjiangensis*] | + | 2.796 |
| UZW38109.1 | transcription termination factor Rho [*Rickettsia conorii* subsp. *heilongjiangensis*] | + | 2.791 |
| UZW39306.1 | histidine--tRNA ligase [*Rickettsia conorii* subsp. *heilongjiangensis*] | + | 2.727 |
| WP_267185174.1 | cell cycle transcriptional regulator TrcR [*Rickettsia conorii*] | + | 2.724 |
| UZW38497.1 | hydrolase [*Rickettsia conorii* subsp. *heilongjiangensis*] | + | 2.722 |
| UZW38572.1 | F0F1 ATP synthase subunit beta [*Rickettsia conorii* subsp. *heilongjiangensis*] | + | 2.716 |
| UZW38356.1 | 50S ribosomal protein L17 [*Rickettsia conorii* subsp. *heilongjiangensis*] | + | 2.708 |
| UZW39013.1 | translational GTPase TypA [*Rickettsia conorii* subsp. *heilongjiangensis*] | + | 2.677 |
| UZW39120.1 | NAD(P)-dependent oxidoreductase [*Rickettsia conorii* subsp. *heilongjiangensis*] | + | 2.676 |
| UZW38842.1 | ribonuclease III [*Rickettsia conorii* subsp. *heilongjiangensis*] | + | 2.634 |
| UZW39250.1 | ADP-forming succinate--CoA ligase subunit beta [*Rickettsia conorii* subsp. *heilongjiangensis*] | + | 2.595 |
| UZW38994.1 | hypothetical protein OSR38_01735 [*Rickettsia conorii* subsp. *heilongjiangensis*] | + | 2.563 |
| UZW38553.1 | serine--tRNA ligase [*Rickettsia conorii* subsp. *heilongjiangensis*] | + | 2.550 |
| UZW38615.1 | porin family protein [*Rickettsia conorii* subsp. *heilongjiangensis*] | + | 2.538 |
| UZW38823.1 | 50S ribosomal protein L28 [*Rickettsia conorii* subsp. *heilongjiangensis*] | + | 2.507 |
| UZW39357.1 | F0F1 ATP synthase subunit alpha [*Rickettsia conorii* subsp. *heilongjiangensis*] | + | 2.501 |
| UZW38658.1 | transcription elongation factor GreA [*Rickettsia conorii* subsp. *heilongjiangensis*] | + | 2.477 |
| UZW38520.1 | DUF5460 family protein [*Rickettsia conorii* subsp. *heilongjiangensis*] | + | 2.419 |
| UZW38199.1 | transcription termination factor NusA [*Rickettsia conorii* subsp. *heilongjiangensis*] | + | 2.391 |
| WP_012736528.1 | MULTISPECIES: universal stress protein [spotted fever group] | + | 2.381 |
| UZW39269.1 | thioredoxin-disulfide reductase [*Rickettsia conorii* subsp. *heilongjiangensis*] | + | 2.329 |
| UZW38854.1 | succinate dehydrogenase flavoprotein subunit [*Rickettsia conorii* subsp. *heilongjiangensis*] | + | 2.249 |
| UZW38979.1 | outer membrane protein assembly factor BamB [*Rickettsia conorii* subsp. *heilongjiangensis*] | + | 2.155 |
| UZW38264.1 | DNA topoisomerase (ATP-hydrolyzing) subunit B [*Rickettsia conorii* subsp. *heilongjiangensis*] | + | 2.155 |
| UZW38859.1 | transcription termination/antitermination protein NusG [*Rickettsia conorii* subsp. *heilongjiangensis*] | + | 2.145 |
| UZW38858.1 | elongation factor G [*Rickettsia conorii* subsp. *heilongjiangensis*] | + | 2.125 |
| UZW38875.1 | ABC transporter substrate-binding protein [*Rickettsia conorii* subsp. *heilongjiangensis*] | + | 2.077 |
| UZW38786.1 | deoxyguanosinetriphosphate triphosphohydrolase [*Rickettsia conorii* subsp. *heilongjiangensis*] | + | 2.076 |
| UZW38340.1 | co-chaperone GroES [*Rickettsia conorii* subsp. *heilongjiangensis*] | + | 2.075 |
| UZW39027.1 | Hsp20/alpha crystallin family protein [*Rickettsia conorii* subsp. *heilongjiangensis*] | + | 1.986 |
| UZW39376.1 | 50S ribosomal protein L7/L12 [*Rickettsia conorii* subsp. *heilongjiangensis*] | + | 1.960 |
| WP_103897598.1 | MULTISPECIES: SurA N-terminal domain-containing protein [spotted fever group] | + | 1.912 |
| WP_267185305.1 | pyruvate dehydrogenase (acetyl-transferring) E1 component subunit alpha [*Rickettsia conorii*] | + | 1.908 |
| UZW38724.1 | DsbA family protein [*Rickettsia conorii* subsp. *heilongjiangensis*] | + | 1.900 |
| UZW38856.1 | 30S ribosomal protein S12 [*Rickettsia conorii* subsp. *heilongjiangensis*] | + | 1.891 |
| UZW38558.1 | class I SAM-dependent methyltransferase [*Rickettsia conorii* subsp. *heilongjiangensis*] | + | 1.886 |
| UZW38339.1 | chaperonin GroEL [*Rickettsia conorii* subsp. *heilongjiangensis*] | + | 1.883 |
| UZW38496.1 | serine hydroxymethyltransferase [*Rickettsia conorii* subsp. *heilongjiangensis*] | + | 1.883 |
| UZW38977.1 | tol-pal system protein YbgF [*Rickettsia conorii* subsp. *heilongjiangensis*] | + | 1.881 |
| UZW39285.1 | bifunctional methylenetetrahydrofolate dehydrogenase/methenyltetrahydrofolate cyclohydrolase FolD [*Rickettsia conorii* subsp. *heilongjiangensis*] | + | 1.768 |
| UZW38504.1 | penicillin-binding protein activator [*Rickettsia conorii* subsp. *heilongjiangensis*] | + | 1.766 |
| UZW38755.1 | 50S ribosomal protein L9 [*Rickettsia conorii* subsp. *heilongjiangensis*] | + | 1.750 |
| UZW38860.1 | 50S ribosomal protein L11 [*Rickettsia conorii* subsp. *heilongjiangensis*] | + | 1.706 |
| UZW38420.1 | phosphorylcholine transferase LicD [*Rickettsia conorii* subsp. *heilongjiangensis*] | + | 1.694 |
| UZW38228.1 | hypothetical protein OSR38_04415 [*Rickettsia conorii* subsp. *heilongjiangensis*] | + | 1.688 |
| AXO78755.1 | outer membrane protein B, partial [*Rickettsia conorii* subsp. *heilongjiangensis*] | + | 1.596 |
| UZW39121.1 | UDP-glucose 4-epimerase [*Rickettsia conorii* subsp. *heilongjiangensis*] | + | 1.589 |
| UZW38330.1 | acyl-CoA carboxylase subunit beta [*Rickettsia conorii* subsp. *heilongjiangensis*] | + | 1.560 |
| UZW38876.1 | Asp-tRNA(Asn)/Glu-tRNA(Gln) amidotransferase subunit GatB [*Rickettsia conorii* subsp. *heilongjiangensis*] | + | 1.554 |
| UZW38085.1 | polyribonucleotide nucleotidyltransferase [*Rickettsia conorii* subsp. *heilongjiangensis*] | + | 1.542 |
| UZW38791.1 | dCTP deaminase [*Rickettsia conorii* subsp. *heilongjiangensis*] | + | 1.510 |
| UZW38380.1 | 50S ribosomal protein L4 [*Rickettsia conorii* subsp. *heilongjiangensis*] | + | 1.419 |
| UZW38793.1 | response regulator transcription factor [*Rickettsia conorii* subsp. *heilongjiangensis*] | + | 1.378 |
| UZW39069.1 | FAD-dependent thymidylate synthase [*Rickettsia conorii* subsp. *heilongjiangensis*] | + | 1.363 |
| WP_267184865.1 | pentapeptide repeat-containing protein [*Rickettsia conorii*] | + | 1.322 |
| UZW38108.1 | class I SAM-dependent methyltransferase [*Rickettsia conorii* subsp. *heilongjiangensis*] | + | 1.317 |
| UZW38383.1 | elongation factor Tu [*Rickettsia conorii* subsp. *heilongjiangensis*] | + | 1.264 |
| WP_045805706.1 | MULTISPECIES: outer membrane protein [spotted fever group] | + | 1.263 |
| WP_267184883.1 | peptidylprolyl isomerase [*Rickettsia conorii*] | + | 1.260 |
| UZW38805.1 | translation elongation factor Ts [*Rickettsia conorii* subsp. *heilongjiangensis*] | + | 1.240 |
| UZW38229.1 | penicillin-binding protein 2 [*Rickettsia conorii* subsp. *heilongjiangensis*] | + | 1.226 |
| UZW38415.1 | methionine--tRNA ligase [*Rickettsia conorii* subsp. *heilongjiangensis*] | + | 1.219 |
| UZW38911.1 | molecular chaperone DnaK [*Rickettsia conorii* subsp. *heilongjiangensis*] | + | 1.215 |
| UZW38348.1 | nucleotide exchange factor GrpE [*Rickettsia conorii* subsp. *heilongjiangensis*] | + | 1.212 |
| UZW38369.1 | 50S ribosomal protein L5 [*Rickettsia conorii* subsp. *heilongjiangensis*] | + | 1.212 |
| UZW38857.1 | 30S ribosomal protein S7 [*Rickettsia conorii* subsp. *heilongjiangensis*] | + | 1.204 |
| UZW39003.1 | hypothetical protein OSR38_01785 [*Rickettsia conorii* subsp. *heilongjiangensis*] | + | 1.084 |
| UZW38262.1 | DNA-directed RNA polymerase subunit omega [*Rickettsia conorii* subsp. *heilongjiangensis*] | + | 1.068 |
| UZW38507.1 | 50S ribosomal protein L21 [*Rickettsia conorii* subsp. *heilongjiangensis*] | + | 1.003 |
| UZW38239.1 | copper chaperone PCu(A)C [*Rickettsia conorii* subsp. *heilongjiangensis*] | + | 0.998 |
| UZW38861.1 | 50S ribosomal protein L1 [*Rickettsia conorii* subsp. *heilongjiangensis*] | + | 0.945 |
| UZW38382.1 | 30S ribosomal protein S10 [*Rickettsia conorii* subsp. *heilongjiangensis*] | + | 0.869 |
| UZW39232.1 | phenylalanine--tRNA ligase subunit beta [*Rickettsia conorii* subsp. *heilongjiangensis*] | + | 0.809 |
| UZW38772.1 | ATP/ADP exchange transporter Tlc1 [*Rickettsia conorii* subsp. *heilongjiangensis*] | + | 0.790 |
| UZW38537.1 | peptidoglycan-associated lipoprotein Pal [*Rickettsia conorii* subsp. *heilongjiangensis*] | + | 0.753 |
| UZW38301.1 | Bcr/CflA family efflux MFS transporter [*Rickettsia conorii* subsp. *heilongjiangensis*] | + | 0.706 |
| UZW38508.1 | 50S ribosomal protein L27 [*Rickettsia conorii* subsp. *heilongjiangensis*] | + | 0.635 |
| UZW38367.1 | 30S ribosomal protein S8 [*Rickettsia conorii* subsp. *heilongjiangensis*] | + | 0.624 |
| UZW38287.1 | Arp2/3 complex-activating protein rickA [*Rickettsia conorii* subsp. *heilongjiangensis*] | + | 0.609 |
| UZW38128.1 | YebC/PmpR family DNA-binding transcriptional regulator [*Rickettsia conorii* subsp. *heilongjiangensis*] |  | 0.582 |
| UZW38862.1 | 50S ribosomal protein L10 [*Rickettsia conorii* subsp. *heilongjiangensis*] |  | 0.560 |
| UZW38096.1 | pyruvate, phosphate dikinase [*Rickettsia conorii* subsp. *heilongjiangensis*] |  | 0.374 |
| UZW38896.1 | HU family DNA-binding protein [*Rickettsia conorii* subsp. *heilongjiangensis*] |  | 0.308 |
| UZW38877.1 | Asp-tRNA(Asn)/Glu-tRNA(Gln) amidotransferase subunit GatA [*Rickettsia conorii* subsp. *heilongjiangensis*] |  | 0.302 |
| UZW38684.1 | 30S ribosomal protein S16 [*Rickettsia conorii* subsp. *heilongjiangensis*] |  | 0.251 |
| UZW39310.1 | peroxiredoxin [*Rickettsia conorii* subsp. *heilongjiangensis*] |  | 0.247 |
| UZW38362.1 | 50S ribosomal protein L15 [*Rickettsia conorii* subsp. *heilongjiangensis*] |  | 0.204 |
| UZW38359.1 | 30S ribosomal protein S13 [*Rickettsia conorii* subsp. *heilongjiangensis*] |  | 0.167 |
| UZW38867.1 | leucyl aminopeptidase [*Rickettsia conorii* subsp. *heilongjiangensis*] |  | 0.117 |
| UZW38181.1 | hypothetical protein OSR38_04145 [*Rickettsia conorii* subsp. *heilongjiangensis*] |  | -0.115 |
| UZW38554.1 | VirB4 family type IV secretion/conjugal transfer ATPase [*Rickettsia conorii* subsp. *heilongjiangensis*] |  | -0.212 |
| UZW38355.1 | 30S ribosomal protein S20 [*Rickettsia conorii* subsp. *heilongjiangensis*] |  | -0.266 |
| UZW38113.1 | hypothetical protein OSR38_03705 [*Rickettsia conorii* subsp. *heilongjiangensis*] |  | -0.317 |
| BBM94850.1 | hypothetical protein RHSENDAI58_02195 [*Rickettsia conorii* subsp. *heilongjiangensis*] | - | -0.703 |
| UZW38824.1 | 50S ribosomal protein L31 [*Rickettsia conorii* subsp. *heilongjiangensis*] | - | -0.732 |
| UZW39268.1 | alpha/beta hydrolase [*Rickettsia conorii* subsp. *heilongjiangensis*] | - | -0.820 |
| UZW38311.1 | 50S ribosomal protein L20 [*Rickettsia conorii* subsp. *heilongjiangensis*] | - | -0.831 |
| UZW38774.1 | nucleoside-diphosphate kinase [*Rickettsia conorii* subsp. *heilongjiangensis*] | - | -0.979 |
| UZW38090.1 | helix-turn-helix transcriptional regulator [*Rickettsia conorii* subsp. *heilongjiangensis*] | - | -1.298 |
| WP_014014694.1 | MULTISPECIES: DUF2673 domain-containing protein [spotted fever group] | - | -1.367 |
| UZW38662.1 | YbaB/EbfC family nucleoid-associated protein [*Rickettsia conorii* subsp. *heilongjiangensis*] | - | -3.806 |
| UZW38373.1 | 50S ribosomal protein L29 [*Rickettsia conorii* subsp. *heilongjiangensis*] | - | -4.795 |
| UZW38326.1 | isoleucine--tRNA ligase [*Rickettsia conorii* subsp. *heilongjiangensis*] | - | -7.451 |
| WP_267185271.1 | ankyrin repeat domain-containing protein [*Rickettsia conorii*] | - | -7.896 |
